# Intracellular microRNA expression patterns influence cell death fates for both necrosis and apoptosis

**DOI:** 10.1002/2211-5463.12995

**Published:** 2020-10-22

**Authors:** Akira Sato, Akihiro Yamamoto, Akira Shimotsuma, Yoko Ogino, Naoki Funayama, Yui Takahashi, Akiko Hiramoto, Yusuke Wataya, Hye‐Sook Kim

**Affiliations:** ^1^ Department of Biochemistry Faculty of Pharmaceutical Sciences Tokyo University of Science Chiba Japan; ^2^ Division of International Infectious Disease Control Faculty of Pharmaceutical Sciences Okayama University Okayama Japan; ^3^ Department of Gene Regulation Faculty of Pharmaceutical Sciences Tokyo University of Science Chiba Japan

**Keywords:** apoptosis, dicer, floxuridine, geldanamycin, microRNA expression, necrosis

## Abstract

MicroRNAs (miRNAs) are small noncoding RNA molecules that interact with target mRNAs at specific sites to induce cleavage of the mRNA or inhibit translation. Such miRNAs play a vital role in gene expression and in several other biological processes, including cell death. We have studied the mechanisms regulating cell death (necrosis in original F28‐7 cells and apoptosis in their variant F28‐7‐A cells) in the mouse mammary tumor cell line FM3A using the anticancer agent floxuridine (FUdR). We previously reported that inhibition of heat‐shock protein 90 by the specific inhibitor geldanamycin (GA) in F28‐7 cells causes a shift from necrosis to apoptosis. In this study, we investigated the intracellular miRNA expression profiles of FUdR‐treated F28‐7 cells (necrotic condition), GA plus FUdR‐treated F28‐7 cells (apoptotic condition), and FUdR‐treated F28‐7‐A cells (apoptotic condition) through miRNA microarray analysis. In addition, we knocked down *Dicer*, a key molecule for the expression of mature miRNAs, in F28‐7 cells to examine whether it modulates FUdR‐induced cell death. Our analysis revealed that the miRNA expression patterns differ significantly between these cell death conditions. Furthermore, we identified miRNA candidates that regulate cell death. Knockdown of *Dicer* in FUdR‐treated necrosis‐fated cells caused a partial shift from necrosis to apoptosis. These findings suggest that modulation of miRNA expression patterns influences the decision of cell death fate toward necrosis or apoptosis. Our findings may serve as a basis for further study of the functions of miRNAs in cell death mechanisms.

## Graphical abstract

We reveal that intracellular miRNA expression patterns are dramatically altered in necrosis and apoptosis using a floxuridine (FUdR)‐induced mouse mammary tumor cell death model. We found that knockdown of *Dicer* in FUdR‐treated necrosis‐fated cells caused a partial shift from necrosis to apoptosis. These findings suggest that the expression of miRNA(s) regulates cell death fate toward necrosis or apoptosis.

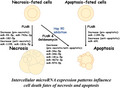

**Abbreviations**
FUdR: floxuridine
GA: geldanamycin
HSP90: heat‐shock protein 90
miRNA: microRNA
siRNA: small interfering RNA

Research on cancer cell death is important to understand the weaknesses of tumors [1]. Numerous previous studies have reported various types of cancer cell death, including apoptosis, necroptosis, parthanatos, and necrosis [2, 3, 4, 5]. We have been investigating the molecular mechanisms regulating necrosis in original F28‐7 cells and apoptosis in their subclone variant F28‐7‐A cells during treatment of mouse mammary carcinoma FM3A cells with floxuridine (5‐fluoro‐2’‐deoxyuridine; FUdR), an anticancer thymidylate synthetase inhibitor [6, 7, 8, 9, 10, 11, 12, 13, 14, 15, 16]. For cell death, two major processes have been characterized according to morphological features, namely necrosis and apoptosis. These two types of cell death after treatment with FUdR, that is, necrosis in F28‐7 cells and apoptosis in F28‐7‐A cells, were recognizable by observing the morphology during cell death [9]. Necrosis in F28‐7 cells is characterized by swelling of the cell and organelles and disruption of cellular and nuclear membranes [9]. Interestingly, inhibition of heat‐shock protein 90 (Hsp90) using the inhibitor geldanamycin (GA) in F28‐7 cells causes a shift from FUdR‐induced necrosis to apoptosis [11]. In addition, necrosis in F28‐7 cells was not suppressed by the necroptosis inhibitor necrostatin‐1. These results indicated necrosis in F28‐7 not necroptosis but other regulatory necrosis [13]. In contrast, apoptosis in F28‐7‐A cells is characterized by membrane blebbing, shrinking of the cell and its organelles, release of cytochrome c from mitochondria, cleavage of caspase‐3 and poly[ADP‐ribose] polymerase 1, and oligonucleosomal degradation of DNA [9]. Previously, we reported six possible regulators in the processes of cell death (necrosis and apoptosis): molecular chaperone Hsp90 [11], nuclear scaffold lamin B1 [10, 12], cytoplasmic intermediate filament cytokeratin‐19 [12], transcription factor activating transcription factor 3 [13], microRNA (miRNA, miR) miR‐351‐5p [14, 15], and miR‐743a‐3p [14]. These cell death regulators were discovered by proteomic and transcriptomic analyses of the cell death model using small interfering RNA (siRNA), miRNA mimics, miRNA inhibitors, or chemical inhibitors. In the present study, we investigated the miRNA expression profiles of FUdR‐induced necrosis in F28‐7 cells, GA plus FUdR‐induced apoptosis in F28‐7 cells, and FUdR‐induced apoptosis in F28‐7‐A cells to understand the molecular mechanisms underlying these two types of cell death (necrosis and apoptosis).

Two major categories of noncoding RNA (ncRNA), that is, miRNA and long noncoding RNA (lncRNA), play important roles in gene expression, cell death, and several physiological processes [17]. The miRNAs are endogenous small ncRNAs (length: 21–25 nucleotides) that function as gene silencers by binding to the specific sites of target mRNAs, inhibiting the initiation of protein synthesis and/or promoting mRNA cleavage [18, 19]. Importantly, miRNAs regulate many biological processes, including cell development, differentiation, and cell death [18, 19, 20, 21]. They are excised in a stepwise process from primary miRNA transcripts [18, 19]. The primary miRNA is cleaved by nuclear RNase III Drosha to release hairpin‐shaped precursor miRNAs (pre‐miRNAs) [18, 19]. These pre‐miRNAs are subsequently exported to the cytoplasm and further processed by Dicer to mature miRNAs [18, 19].

In this study, we showed that the intracellular miRNA expression patterns were dramatically altered in cell death (necrosis and apoptosis) using the cell death model. We found that knockdown of *Dicer* in FUdR‐treated necrosis‐fated cells caused a partial shift from necrosis to apoptosis. These findings suggest that the expression of miRNA(s) regulates cell death fate toward necrosis or apoptosis.

## Materials and methods

### Reagents

Floxuridine and GA were obtained from Sigma (St. Louis, MO, USA). FUdR was stored as 2 mm stocks in ultrapure water at −20 °C. GA was stored as 2 mm stock in dimethyl sulfoxide at −20 °C with protection from light. 4’,6‐Diamidino‐2‐phenylindole dihydrochloride was obtained from Invitrogen (Carlsbad, CA, USA).

### Cell culture

Mouse mammary tumor FM3A cells (original F28‐7 clone and variant F28‐7‐A clone) were maintained by suspension culture [9, 13, 14]. These cells were grown in ES medium containing 2% heat‐inactivated FBS at 37 °C under a humidified 5% CO_2_ atmosphere. F28‐7 and F28‐7‐A cells (~ 2 × 10^5^ cells per mL) were treated with 1 μm FUdR. Cell viability was estimated with a hemocytometer by means of trypan blue exclusion.

### RNA extraction

RNA extraction was performed as previously described [10, 11, 14]. For microarray analysis, the total RNA fraction was isolated from the individual cell lines using QIAshredder spin columns and an RNeasy Mini Kit, according to the instructions provided by the manufacturer (QIAGEN, Hilden, Germany).

### Microarray analysis of miRNA expression

Microarray analysis of miRNA expression was performed as previously described [14]. Biotin‐labeled RNA was prepared using a FlashTag™ Biotin RNA Labeling Kit according to the instructions provided by the manufacturer (Genisphere, Hatfield, PA, USA). The labeled RNA product was mixed in hybridization cocktail. The hybridization cocktails were added to GeneChip® miRNA 2.0 Array (Affymetrix, Santa Clara, CA, USA) in the GeneChip Hybridization Oven 640 under constant rotation (60 r.p.m.) at 48 °C for 16 h. After the hybridization, GeneChips were washed and stained with GeneChip Fluidics Station 450 (Affymetrix) and scanned with a GeneChip Scanner 3000 7G (Affymetrix). Scanned GeneChip images were analyzed using the Affymetrix GeneChip Command Console software and miRNA QCTool (Affymetrix). Microarray data were analyzed using the genespring software (Agilent Technologies, Santa Clara, CA, USA).

### *Dicer*‐targeted siRNA transfection

Transfection of *Dicer*‐targeted or nonsilencing siRNA (siNS) was performed by electroporation as previously described [10, 12, 13]. A set of four siRNAs against *Dicer* was used, namely Mm_Dicer1_1 (catalog number, SI00979335), Mm_Dicer1_2 (catalog number, SI00979342), Mm_Dicer1_3 (catalog number, SI00979349), and Mm_Dicer1_6 (catalog number, SI02747101). AllStars Negative Control siRNA (catalog number, 1027280) was used as siNS. These siRNAs were obtained from QIAGEN. The *Dicer*‐targeted siRNA mixture was prepared by combining the *Dicer*‐targeted four siRNAs. In the 0.1‐cm‐gap cuvette, F28‐7 cells (2 × 10^5^ cells) were suspended in 75 μL siPORT electroporation buffer (Ambion, Austin, TX, USA), containing *Dicer*‐targeted siRNA mixture or siNS (final concentration: 800 nm). Subsequently, cells were electroporated using the Gene Pulser Xcell (Bio‐Rad, Hercules, CA, USA) at voltage of 0.15 kV, pulse length of 1000 μs, and number of pulse of 1.

### Real‐time PCR analysis

Quantitative real‐time PCR analysis of mRNA expression was performed as previously described [13]. Firstly, cDNA samples were reverse‐transcribed using 1.5 μg total RNA, an oligo(dT) primer (Invitrogen), and Ready‐To‐Go You‐Prime First‐Strand Beads (GE Healthcare, Chicago, IL, USA). The mRNA expression levels of *Dicer* were analyzed using LightCycler FastStart DNA Master SYBR Green I and a LightCycler (Roche, Basel, Switzerland). The levels of mRNA were normalized using *Gapdh* mRNA and quantified using the comparative cycle threshold method. The primers used for the *Dicer* were 5′‐CTTGACTGACTTGCGCTCTG‐3′ (forward primer) and 5′‐AATGGCACCAGCAAGAGACT‐3′ (reverse primer). The primers used for the *Gapdh* were 5′‐ATCACCATCTTCCAGGAGCGAGAAAT‐3′ (forward primer) and 5′‐ATGCCAGTGAGCTTCCCGTTCAG‐3′ (reverse primer). These primers were synthesized by Hokkaido Bio System (Hokkaido, Japan).

### Observation of cell morphology

The observation of cell morphology was performed as previously described [13, 14] using an Olympus (Tokyo, Japan) BX61 fluorescence microscope.

### Statistical analysis

Determining the statistical significance of differences among groups was conducted using the Student's *t*‐test and one‐way analysis of variance. *P* < 0.05 denoted statistically significant differences.

## Results and Discussion

### The miRNA expression profiles of necrosis in F28‐7 cells and apoptosis in F28‐7‐A cells after treatment with FUdR

**Fig. 1:**
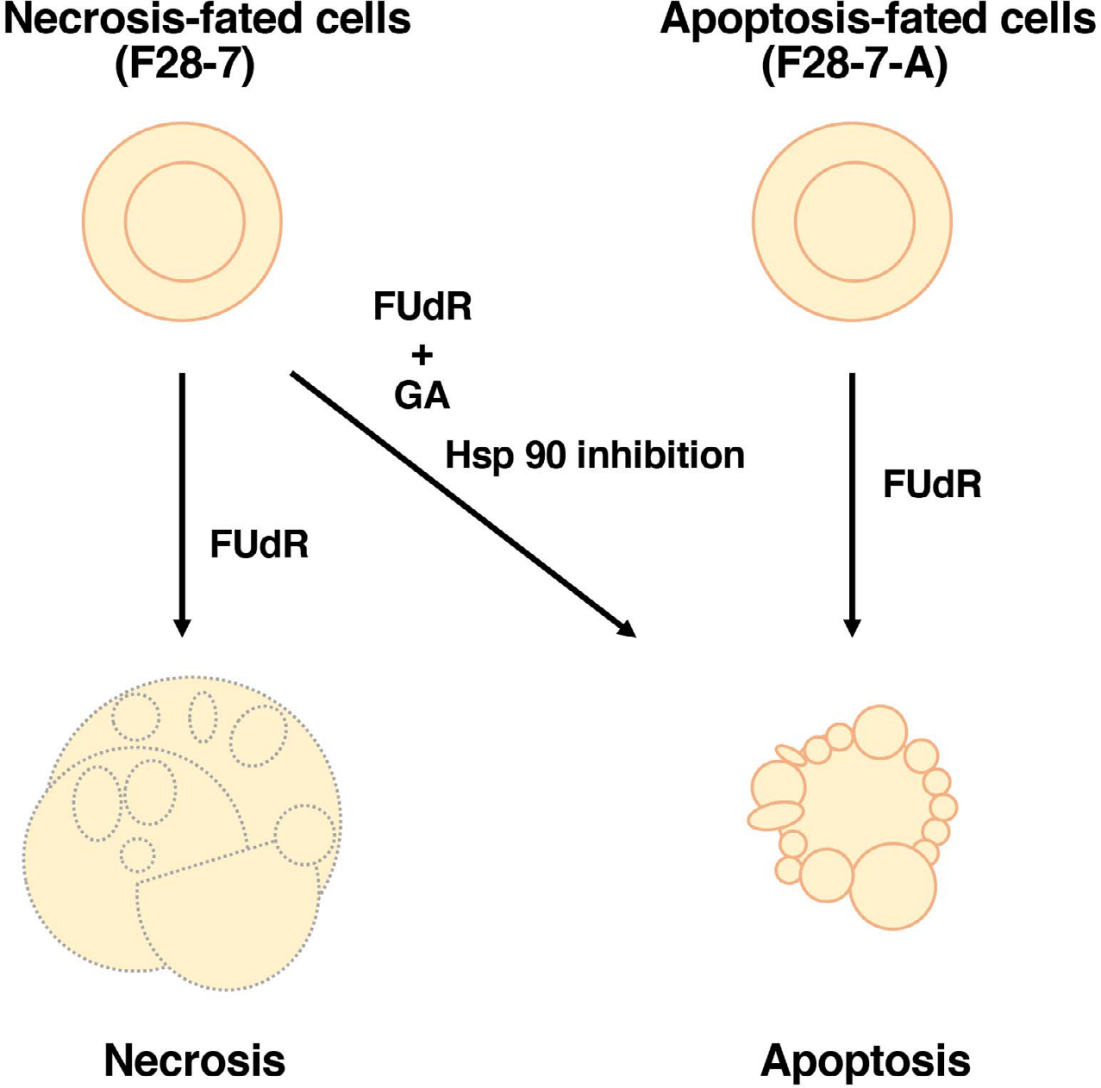
FUdR‐induced necrosis and apoptosis using the cell death model. F28‐7 cells undergoing necrosis after treatment with FUdR. F28‐7‐A cells undergoing apoptosis after treatment with FUdR. Apoptosis induced in F28‐7 cells after treatment with the combination of GA and FUdR.

F28‐7 and F28‐7‐A cells were treated with 1 μm FUdR for 0 h (no drug, no incubation) and 8 h to profile changes in the pattern of miRNA expression in FUdR‐induced necrosis and apoptosis. The treatment induced necrosis in F28‐7 cells and apoptosis in F28‐7‐A cells (Fig. 1) [9, 11]. We investigated the miRNA expression profiles in necrotic and apoptotic dying cells through miRNA microarray analysis. For this purpose, three independent analyses were performed with the Affymetrix GeneChip miRNA 2.0 Array, which contains a 722‐probe set for mouse mature miRNAs and 690‐probe set for mouse pre‐miRNA, allowing analyses of the expression levels of mouse mature miRNAs. Previously, we reported the miRNA expression profiles in these sister cells at the untreated stage (no drug) by microarray analyses [14]. We analyzed the differentially expressed miRNAs in the dying stage vs the untreated stage in both F28‐7 and F28‐7‐A cells. Using a 1.5‐fold cutoff (*P* < 0.05), the analysis identified 14 differentially expressed miRNAs. In necrosis (FUdR‐treated F28‐7 cells), 3 and 11 mature miRNAs were expressed at higher and lower levels, respectively, than in the untreated stage (F28‐7 cells). Table 1 lists the names and sequence information of these 14 mature miRNAs. The miRNA expression analysis identified two differentially expressed miRNAs. In apoptosis (FUdR‐treated F28‐7‐A cells), two mature miRNAs (i.e., miR‐1199‐5p and miR‐691) were expressed at higher and lower levels, respectively, than in the untreated stage (F28‐7‐A cells). Table 2 lists the names and sequence information of these two mature miRNAs.

**Table 1:** Differentially expressed miRNAs in necrosis‐fated F28‐7 cells. FC, fold change (FUdR 8 h/ 0 h in F28‐7 cells)> 1.5 or < 0.7 (indicates ≤ 0.67), *P* < 0.05.

miRNA name	FC (F8h vs 0h)	Sequence
Upregulated miRNAs in FUdR‐treated F28‐7 cells		
mmu‐miR‐93‐5p	1.7	CAAAGUGCUGUUCGUGCAGGUAG
mmu‐miR‐743a‐3p	1.5	GAAAGACACCAAGCUGAGUAGA
mmu‐miR‐182‐5p	1.5	UUUGGCAAUGGUAGAACUCACACCG
Downregulated miRNAs in FUdR‐treated F28‐7 cells		
mmu‐miR‐466j	0.7	UGUGUGCAUGUGCAUGUGUGUAA
mmu‐let‐7a‐5p	0.7	UGAGGUAGUAGGUUGUAUAGUU
mmu‐miR‐331‐3p	0.6	GCCCCUGGGCCUAUCCUAGAA
mmu‐miR‐1195	0.6	UGAGUUCGAGGCCAGCCUGCUCA
mmu‐miR‐181b‐5p	0.6	AACAUUCAUUGCUGUCGGUGGGU
mmu‐miR‐155‐5p	0.6	UUAAUGCUAAUUGUGAUAGGGGU
mmu‐miR‐466g	0.6	AUACAGACACAUGCACACACA
mmu‐miR‐466f	0.6	ACGUGUGUGUGCAUGUGCAUGU
mmu‐miR‐296‐3p	0.5	GAGGGUUGGGUGGAGGCUCUCC
mmu‐miR‐705	0.4	GGUGGGAGGUGGGGUGGGCA
mmu‐miR‐712‐5p	0.3	CUCCUUCACCCGGGCGGUACC

**Table 2:** Differentially expressed miRNAs in apoptosis‐fated F28‐7‐A cells. FC, fold change (FUdR 8 h/0 h in F28‐7‐A cells)> 1.5 or < 0.7 (indicates ≤ 0.67), *P* < 0.05.

miRNA name	FC (F8h vs 0h)	Sequence
Upregulated miRNA in FUdR‐treated F28‐7‐A cells		
mmu‐miR‐1199‐5p	1.5	UCUGAGUCCCGGUCGCGCGG
Downregulated miRNA in FUdR‐treated F28‐7‐A cells		
mmu‐miR‐691	0.6	AUUCCUGAAGAGAGGCAGAAAA

### The miRNA expression profiles of apoptosis in F28‐7 cells after cotreatment with GA and FUdR

Previously, we also demonstrated that inhibition of Hsp90 by the Hsp90 inhibitor GA in F28‐7 cells causes a shift from original necrosis to apoptosis (Fig. 1) [11]. We investigated the miRNA expression profiles of original necrosis in F28‐7 cells treated with FUdR alone and apoptosis in F28‐7 cells treated with the combination of GA and FUdR by miRNA microarray analysis. Using a 1.5‐fold cutoff, the analysis identified 25 differentially expressed miRNAs. In apoptosis (GA‐ and FUdR‐treated F28‐7 cells), 2 and 23 mature miRNAs were expressed at higher and lower levels, respectively, than in the untreated stage (F28‐7 cells). Table 3 lists the names and sequence information of these 25 mature miRNAs. As shown in Table 4, the analysis identified 24 differentially expressed miRNAs in GA‐treated F28‐7 cells compared with untreated F28‐7 cells.

**Table 3:** Differentially expressed miRNAs in apoptosis‐fated F28‐7 cells. FC, fold change (FUdR and GA 8 h/ 0 h in F28‐7 cells)> 1.5 or < 0.7 (indicates ≤ 0.67), *P* < 0.05.

miRNA name	FC (FG8h vs 0h)	Sequence
Upregulated miRNAs in FUdR‐ and GA‐treated in F28‐7 cells		
mmu‐miR‐690	13.4	AAAGGCUAGGCUCACAACCAAA
mmu‐miR‐16‐5p	1.8	UAGCAGCACGUAAAUAUUGGCG
Downregulated miRNAs in FUdR‐ and GA‐treated in F28‐7 cells		
mmu‐miR‐155‐5p	0.7	UUAAUGCUAAUUGUGAUAGGGGU
mmu‐miR‐34a‐5p	0.6	UGGCAGUGUCUUAGCUGGUUGU
mmu‐miR‐331‐3p	0.6	GCCCCUGGGCCUAUCCUAGAA
mmu‐miR‐466j	0.6	UGUGUGCAUGUGCAUGUGUGUAA
mmu‐miR‐125b‐5p	0.6	UCCCUGAGACCCUAACUUGUGA
mmu‐miR‐346‐5p	0.6	UGUCUGCCCGAGUGCCUGCCUCU
mmu‐miR‐15b‐5p	0.6	UAGCAGCACAUCAUGGUUUACA
mmu‐miR‐181a‐5p	0.6	AACAUUCAACGCUGUCGGUGAGU
mmu‐miR‐351‐5p	0.5	UCCCUGAGGAGCCCUUUGAGCCUG
mmu‐miR‐467d‐3p	0.5	AUAUACAUACACACACCUACAC
mmu‐miR‐466f	0.5	ACGUGUGUGUGCAUGUGCAUGU
mmu‐miR‐34c‐3p	0.5	AAUCACUAACCACACAGCCAGG
mmu‐miR‐296‐3p	0.5	GAGGGUUGGGUGGAGGCUCUCC
mmu‐miR‐125a‐5p	0.4	UCCCUGAGACCCUUUAACCUGUGA
mmu‐miR‐183‐5p	0.4	UAUGGCACUGGUAGAAUUCACU
mmu‐miR‐466f‐5p	0.4	UACGUGUGUGUGCAUGUGCAUG
mmu‐miR‐193a‐5p	0.4	UGGGUCUUUGCGGGCAAGAUGA
mmu‐miR‐466g	0.4	AUACAGACACAUGCACACACA
mmu‐miR‐574‐5p	0.4	UGAGUGUGUGUGUGUGAGUGUGU
mmu‐miR‐712‐5p	0.4	CUCCUUCACCCGGGCGGUACC
mmu‐miR‐714	0.3	CGACGAGGGCCGGUCGGUCGC
mmu‐miR‐466f‐3p	0.3	CAUACACACACACAUACACAC
mmu‐miR‐705	0.3	GGUGGGAGGUGGGGUGGGCA

**Table 4:** Differentially expressed miRNAs in GA‐treated F28‐7 cells. FC, fold change (GA 8 h/0 h in F28‐7 cells)> 1.5 or < 0.7 (indicates ≤ 0.67), *P* < 0.05.

miRNA name	FC (G8h vs 0h)	Sequence
Upregulated miRNAs in GA‐treated F28‐7 cells		
mmu‐miR‐690	15.3	AAAGGCUAGGCUCACAACCAAA
mmu‐miR‐16‐5p	2.3	UAGCAGCACGUAAAUAUUGGCG
mmu‐miR‐669g	1.6	UGCAUUGUAUGUGUUGACAUGAU
Downregulated miRNAs in GA‐treated F28‐7 cells		
mmu‐miR‐27b‐3p	0.7	UUCACAGUGGCUAAGUUCUGC
mmu‐miR‐125b‐5p	0.6	UCCCUGAGACCCUAACUUGUGA
mmu‐let‐7d‐3p	0.6	CUAUACGACCUGCUGCCUUUCU
mmu‐miR‐193a‐5p	0.6	UGGGUCUUUGCGGGCAAGAUGA
mmu‐miR‐34c‐3p	0.6	AAUCACUAACCACACAGCCAGG
mmu‐let‐7a‐5p	0.6	UGAGGUAGUAGGUUGUAUAGUU
mmu‐miR‐351‐5p	0.5	UCCCUGAGGAGCCCUUUGAGCCUG
mmu‐miR‐207	0.5	GCUUCUCCUGGCUCUCCUCCCUC
mmu‐miR‐712‐5p	0.5	CUCCUUCACCCGGGCGGUACC
mmu‐miR‐296‐3p	0.5	GAGGGUUGGGUGGAGGCUCUCC
mmu‐miR‐155‐5p	0.5	UUAAUGCUAAUUGUGAUAGGGGU
mmu‐miR‐1187	0.5	UAUGUGUGUGUGUAUGUGUGUAA
mmu‐miR‐744‐5p	0.5	UGCGGGGCUAGGGCUAACAGCA
mmu‐miR‐15b‐5p	0.5	UAGCAGCACAUCAUGGUUUACA
mmu‐miR‐714	0.5	CGACGAGGGCCGGUCGGUCGC
mmu‐miR‐466g	0.5	AUACAGACACAUGCACACACA
mmu‐miR‐125a‐5p	0.4	UCCCUGAGACCCUUUAACCUGUGA
mmu‐miR‐466f‐5p	0.4	UACGUGUGUGUGCAUGUGCAUG
mmu‐miR‐183‐5p	0.4	UAUGGCACUGGUAGAAUUCACU
mmu‐miR‐705	0.3	GGUGGGAGGUGGGGUGGGCA
mmu‐miR‐466f‐3p	0.3	CAUACACACACACAUACACAC

### Association with miRNA expression and two types of cancer cell death modes

**Fig. 2:**
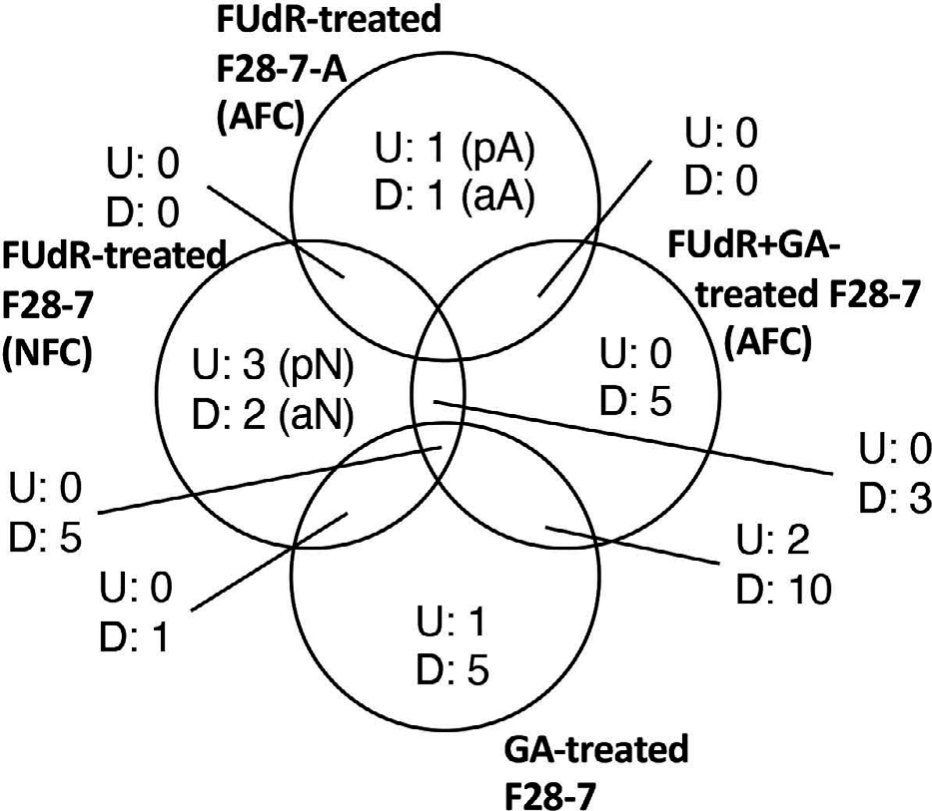
Comparative analyses of the miRNA microarray data. Venn diagram of the microarray data from FUdR‐treated F28‐7‐A cells (apoptotic dying cell condition), FUdR‐treated F28‐7 cells (necrotic dying cell condition), GA plus FUdR‐treated F28‐7 cells (apoptotic dying cell condition), and GA‐treated F28‐7 cells. U, upregulated miRNA; D, downregulated miRNA; pN, pro‐necrotic; aN, anti‐necrotic; pA, pro‐apoptotic; aA, anti‐apoptotic.

Two major members of ncRNA, namely miRNA and lncRNA, play important regulatory roles in gene expression and numerous important physiological processes, which include cell death [17]. Several reports indicated that ncRNA, including miRNA and lncRNA, function as prodeath or antideath signals in various cell death modes, that is, apoptosis and programmed necrosis as necroptosis [17]. We identified necrosis‐related or apoptosis‐related miRNAs by Venn diagram of the microarray data from FUdR‐treated F28‐7‐A cells (apoptotic dying cell condition), FUdR‐treated F28‐7 cells (necrotic dying cell condition), GA plus FUdR‐treated F28‐7 cells (apoptotic dying cell condition), and GA‐treated F28‐7 cells (Fig. 2). Analysis showed the presence of necrosis‐related miRNA candidates (pro‐necrotic: miR‐93‐5p, miR‐743a‐3p, and miR‐182‐5p and anti‐necrotic: miR‐1195 and miR‐181b‐5p) and apoptosis‐related miRNAs candidates (pro‐apoptotic: miR‐1199‐5p and anti‐apoptotic; miR‐691). We will further examine the roles of these cell death‐related miRNAs in the cell death mechanisms of necrosis or apoptosis using synthetic miRNA mimics and miRNA inhibitors. Importantly, apoptosis‐related miRNAs were not shared by two types of apoptotic dying condition under the FUdR‐treated F28‐7‐A and GA plus FUdR‐treated F28‐7 cells (Fig. 2). This finding suggests that the cell death fate of necrosis and apoptosis is determined by a miRNA expression patterns of the normal cell condition (untreated condition) or the early stages (within 8h after FUdR treatment) on cell death mode.

We previously reported that two unique miRNAs (i.e., miR‐351‐5p and miR‐743a‐3p) may regulate the processes of cell death, necrosis, and apoptosis [14]. These miRNAs exhibited higher expression in apoptotic cells (F28‐7‐A) than in necrotic cells (F28‐7). Transfection of a miR‐351‐5p or miR‐743a mimic in necrosis‐fated F28‐7 cells resulted in a shift of the cell death mode from necrosis to apoptosis [14]. In addition, we found that miR‐351‐5p regulated the expression of nuclear scaffold lamin B1, its previously identified regulator of cell death [10, 12], and mediated FUdR‐induced apoptosis [14]. Recently, we showed that miR‐351‐5p directly interacts with a lamin B1 mRNA using the cell‐free *in vitro* miRNA and mRNA‐binding evaluation system [15]. We considered that both miR‐351‐5p and miR‐743a‐3p function as pro‐apoptotic miRNAs in the cell death mechanisms. Interestingly, miR‐351‐5p was downregulated by 0.5‐fold after cotreatment with GA and FUdR vs the untreated condition (Table 3). Also, miR‐743a‐3p was upregulated by 1.5‐fold after treatment with FUdR vs the untreated condition (Table 1). These data suggest that the expression of cell death regulators miR‐351‐5p and miR‐743a‐3p plays an important role in the decision of cell death fate under normal condition. Furthermore, the expression of miR‐351‐5p and miR‐743a‐3p may be important very early in the cell death fate‐determining process. Notably, *Dicer* is the predicted target of miR‐351‐5p in miRDB (http://mirdb.org/index.html).

Through this miRNA microarray analysis, we revealed that the intracellular miRNA expression patterns differ between FUdR‐treated F28‐7 cells (necrotic dying cell condition), GA plus FUdR‐treated F28‐7 cells (apoptotic dying cell condition), and FUdR‐treated F28‐7‐A cells (apoptotic dying cell condition). Interestingly, we revealed that miR‐1199‐5p (a pro‐apoptotic miRNA candidate) is specifically upregulated by 1.5‐fold after treatment with FUdR in F28‐7‐A cells (i.e., under the apoptotic dying cell condition) vs the untreated condition (Table 2). Of note, the roles of miR‐1199‐5p in cell death mechanisms are poorly understood. Diepenbruck et al. reported that miR‐1199‐5p acts as a repressor of epithelial–mesenchymal transition, tumor migration, invasion, and lung metastasis [22]. In addition, mechanistically, miR‐1199‐5p functions in a reciprocal double‐negative feedback loop with the epithelial–mesenchymal transition transcription factor zinc finger E‐box‐binding homeobox 1 (Zeb1) [22]. We will further investigate the functions of miR‐1199‐5p in the regulatory mechanisms of necrosis–apoptosis switching using synthetic miRNA mimics and miRNA inhibitors. Of note, Calabrese et al. reported that the expression of miR‐1199‐5p is affected by knockdown of *Dicer* (a miRNA processing‐related RNase III‐like enzyme) in mouse embryonic stem (ES) cells [23]. In addition, knockdown of *Dicer* induces alterations in the expression pattern of miRNAs in mouse ES cells [23].

### Modulation of FUdR‐induced necrosis by silencing of *Dicer* expression

**Fig. 3:**
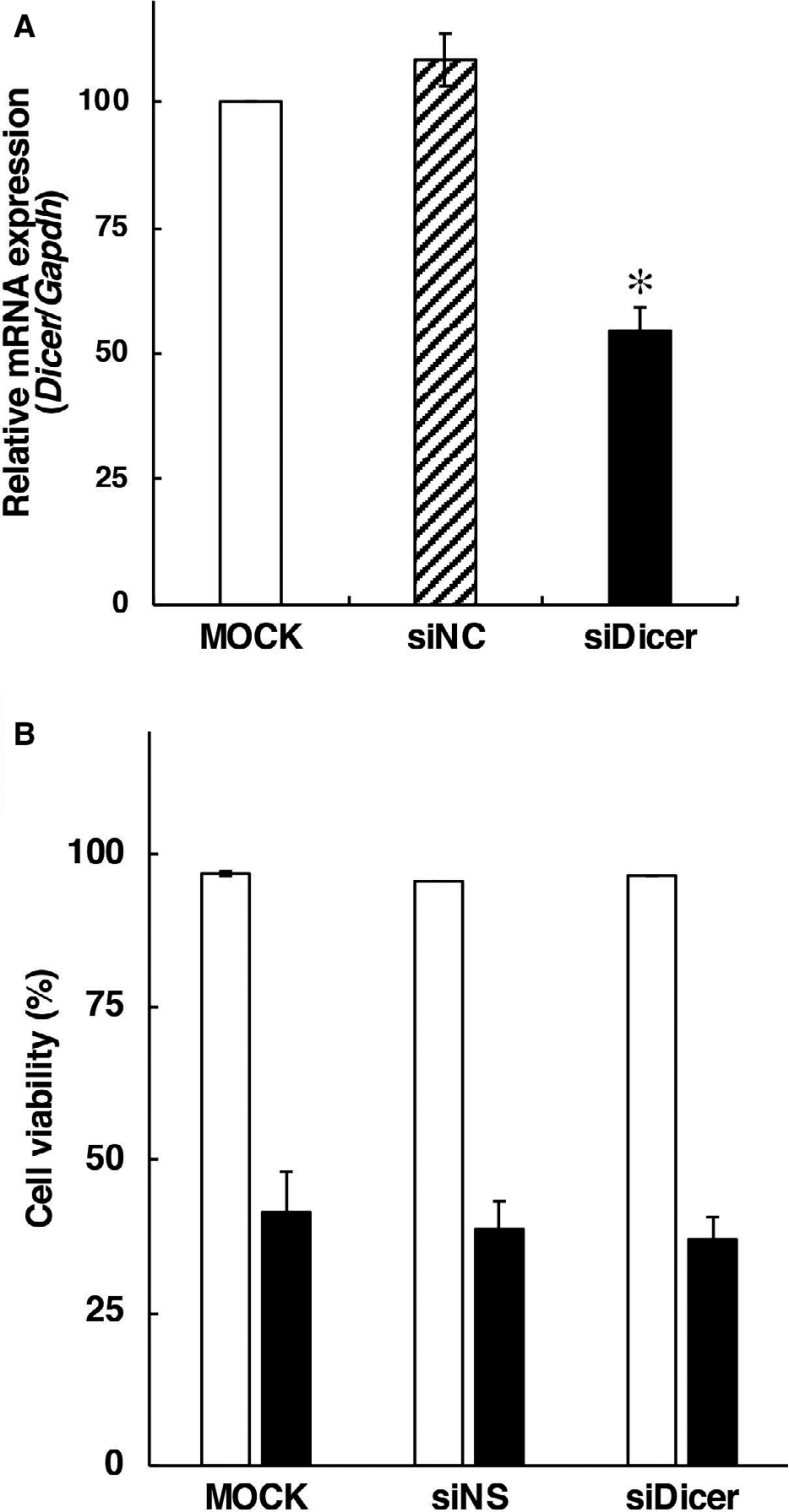
Downregulation of *Dicer* mRNA by transfection of specific *Dicer*‐targeted siRNAs. (A) At 48 h after transfection, the mRNA expression levels of *Dicer* and *Gapdh* (internal control) were analyzed by quantitative real‐time PCR. In the knockdown experiments, F28‐7 cells were transfected with buffer alone (vehicle), siNS, or *Dicer*‐targeted siRNA mixture (siDicer). Results are the mean of three independent experiments with error bars showing the ± SE. * indicates *t*‐test *P* < 0.05 (vs siNC) and one‐way ANOVA *P* = 5.096 × 10^−4^. (B) At 48 h after transfection, F28‐7 cells were treated with 1 μm FUdR for 0 h (no FUdR, no incubation, white bars) or 21 h (black bars). The cell viability was examined by trypan blue dye exclusion. Values are the mean ± SE of three independent experiments. One‐way ANOVA, *P* = 1.298 × 10^−12^.

**Fig. 4:**
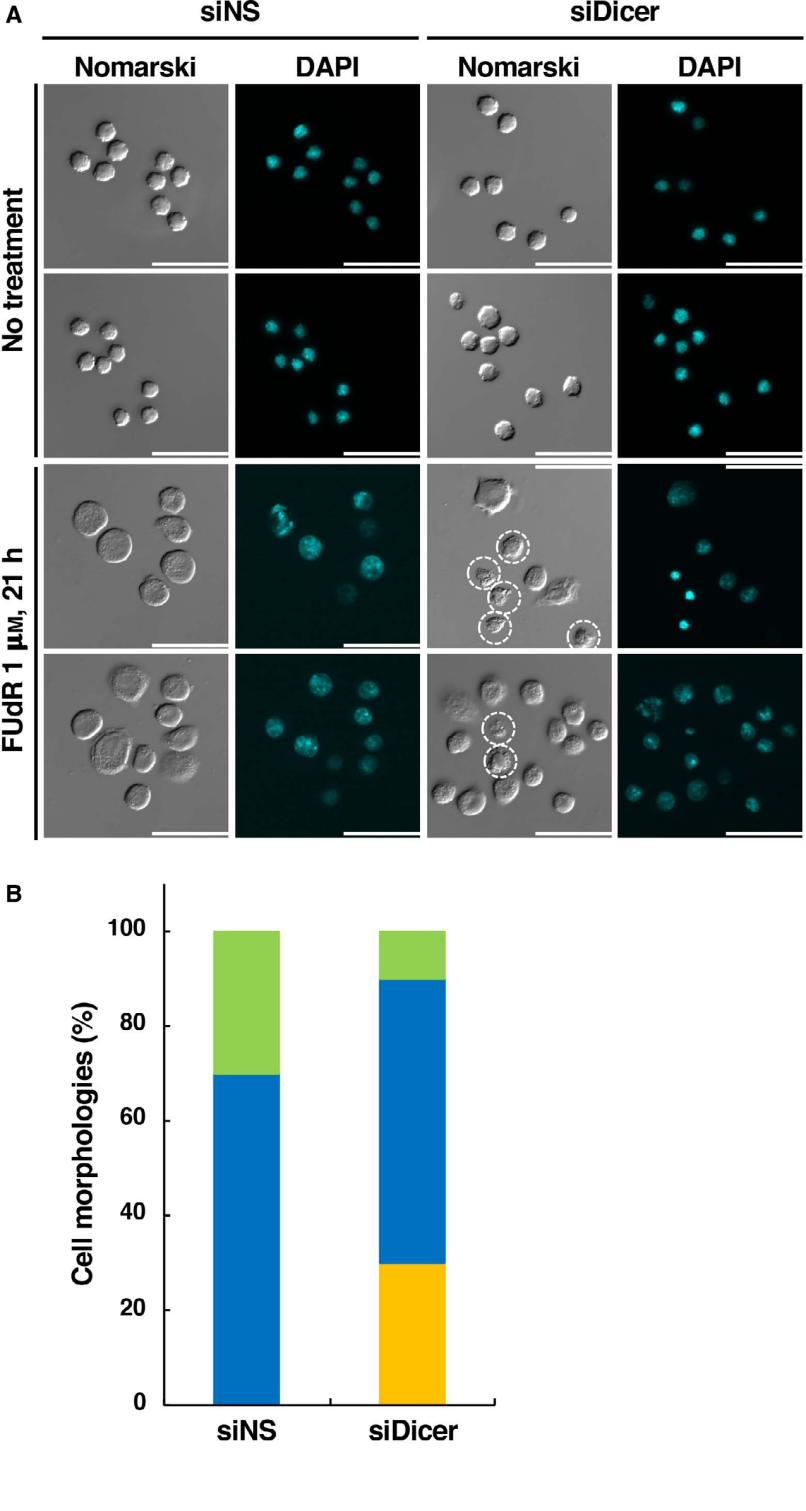
Knockdown of *Dicer* shifts the FUdR‐induced necrosis to apoptosis. (A) At 48 h after transfection with siNS or *Dicer*‐targeted siRNA mixture (siDicer), F28‐7 cells were treated with or without 1 μm FUdR for 21 h and subsequently stained with 4′,6‐diamidino‐2‐phenylindole dihydrochloride as described in the Experimental Procedures section. Cell morphology was analyzed through fluorescence microscopy (objective ×40). The dotted line circle indicates apoptotic cell morphologies. Scale bar = 50 μm. (B) Percentage of necrosis (blue bars), apoptosis (orange bars), and normal cell morphologies (green bars) in FUdR‐treated F28‐7 cells transfected with siNS or *Dicer*‐targeted siRNA mixture (siDicer).

Previous studies have shown that some RNase III‐like enzymes, such as Drosha and Dicer, are involved in miRNA biogenesis [18, 19]. Especially, pre‐miRNAs are cleaved by the RNase III‐like enzyme Dicer to generate mature miRNAs [18, 19]. We investigated the association with the expression of miRNAs and FUdR‐induced cell death modes. We performed transfection of the *Dicer*‐targeted specific siRNA mixture to examine whether gene silencing of *Dicer* (a key molecule for the expression of mature miRNAs) in F28‐7 cells modulates FUdR‐induced cell death. Quantitative real‐time PCR analysis was performed for the RNA fraction in F28‐7 cells at 48 h after the transfection of electroporation buffer alone, siNS, or *Dicer*‐targeted siRNAs (Fig. 3A). The mRNA expression levels of *Dicer* in F28‐7 cells were reduced < 50 % in the *Dicer*‐targeted siRNA‐transfected cells compared with those observed in the siNS ‐transfected cells. As shown in Fig. 3B, transfection of either siNS or *Dicer*‐targeted siRNAs in F28‐7 cells did not change the cell viability after treatment with or without FUdR. We examined the cell morphology of F28‐7 cells with low expression of *Dicer* after treatment with FUdR. Transfection alone of the siNS or the *Dicer*‐targeted RNAs did not have an impact on the cell morphology (Fig. 4A, upper diagram). The necrotic cell morphology in F28‐7 cells and apoptotic cell morphology in F28‐7‐A cells were characteristically observed 21 h after treatment with FUdR [9]. At that time point, the transfection of siNS in F28‐7 cells showed a typical necrotic cell feature, namely cell swelling. In contrast, transfection of *Dicer*‐targeted siRNAs in F28‐7 cells partially showed typical apoptotic cell features, namely cell blebbing and chromatin condensation (Fig. 4A, bottom). The microscopic observation showed that the distribution of cell morphologies was 10% normal, 60% necrosis, and 30% apoptosis in the *Dicer*‐siRNA‐transfected F28‐7 cells after treatment with FUdR (Fig. 4B). In the control experiment, the transfection of siNS in F28‐7 cells showed that the fraction of cell morphologies was 30% normal and 70% necrosis after treatment with FUdR (Fig. 4B). We further elucidate the regulatory mechanisms of the cell death switch from necrosis to apoptosis and the altered miRNA expression by the *Dicer* knockdown. Our findings suggest that gene silencing of *Dicer* partially shifts FUdR‐induced necrosis to apoptosis, indicating that the modulation of miRNA expression patterns influences the determination of cell death fate toward necrosis or apoptosis.

## Conclusions

Using the FUdR‐induced cell death model, we revealed that the expression patterns of miRNAs differ between necrosis and apoptosis. The alterations in the miRNA expression pattern by knockdown of *Dicer* in necrosis‐fated cells changed the cell death mode from necrosis to apoptosis. The present findings may be important in studying the functions of ncRNAs in cell death mechanisms.

## Conflict of interest

The authors declare no conflict of interest.

## Author contributions

AS (Akira Sato) conceived and designed the project. AS (Akira Sato) and AY acquired the data. AS (Akira Sato), AY, AS (Akira Shimotsuma), YO, NF, YT, AH, YW, and HSK analyzed and interpreted the data. AS (Akira Sato) wrote the paper.

## Data Availability

All data will be available from the corresponding author upon reasonable request.
